# The Effect of 8,5′-Cyclo 2′-deoxyadenosine on the Activity of 10-23 DNAzyme: Experimental and Theoretical Study

**DOI:** 10.3390/ijms25052519

**Published:** 2024-02-21

**Authors:** Marcin Cieślak, Bolesław T. Karwowski

**Affiliations:** Food Science Department, Faculty of Pharmacy, Medical University of Lodz, ul. Muszynskiego 1, 90-151 Lodz, Poland; marcin.cieslak@umed.lodz.pl

**Keywords:** 8,5′-cyclo 2′-deoxyadenosine, DNAzyme 10-23, catalytic DNA, molecular dynamics, DNA damage

## Abstract

The in vivo effectiveness of DNAzymes 10-23 (Dz10-23) is limited due to the low concentration of divalent cations. Modifications of the catalytic loop are being sought to increase the activity of Dz10-23 in physiological conditions. We investigated the effect of 5′S or 5′R 5′,8-cyclo-2′deoxyadenosine (cdA) on the activity of Dz10-23. The activity of Dz10-23 was measured in a cleavage assay using radiolabeled RNA. The Density Functional Tight Binding methodology with the self-consistent redistribution of Mulliken charge modification was used to explain different activities of DNAzymes. The substitution of 2′-deoxyadenosine with cdA in the catalytic loop decreased the activity of DNAzymes. Inhibition was dependent on the position of cdA and its absolute configuration. The order of activity of DNAzymes was as follows: wt-Dz > *S*cdA5-Dz ≈ *R*cdA15-Dz ≈ *S*cdA15-Dz > *R*cdA5-Dz. Theoretical studies revealed that the distance between phosphate groups at position 5 in *R*cdA5-Dz was significantly increased compared to wt-Dz, while the distance between O4 of dT4 and nonbonding oxygen of PO_2_ attached to 3′O of dG2 was much shorter. The strong inhibitory effect of RcdA5 may result from hampering the flexibility of the catalytic loop (increased rigidity), which is required for the proper positioning of Me^2+^ and optimal activity.

## 1. Introduction

DNAzymes (Dz) are short, single-stranded DNA molecules endowed with a specific catalytic activity. They do not exist in nature but were developed in an in vitro selection approach (SELEX). Dz catalyse a number of biochemical reactions, for example, the cleavage of RNA [1], the photolysis of C-C bonds in cyclobutane pyrimidine dimers [2], or the ligation of DNA [3]. However, their catalytic repertoire is much wider, including DNA cleavage, esterase- and phosphatase-like activities, C-C bond formation, the ligation of RNA and porphyrin metalation. An extensive review of the catalytic potential of Dz is presented in [4]. Dz10-23 is an example of the RNA-cleaving DNAzyme, which was selected by Santoro and Joyce as clone 23 in the 10th round of the SELEX procedure [5]. Dz10-23 shows high catalytic efficiency (k_cat_/K_M_ ~10^9^ M^−1^ · min^−1^) and is composed of a central 15 nt fragment (catalytic loop that cleaves the internucleotide bond), which is flanked by sequences recognising and binding RNA substrate through Watson–Crick base pairing. Divalent metal ions play a critical role in the activity of Dz10-23. The results of crystallographic and NMR studies suggest that there are three metal-ion binding sites within the Dz10-23/substrate RNA complex. Two of them are assembled by nucleotides composing the catalytic loop, i.e., nucleotides 1–6 at the 5′ end of the loop and nucleotides 12–15 at the 3′ end of the loop. Interestingly, positions 5 and 15 contain 2′-deoxyadenosine (dA), highlighting the importance of this deoxynucleoside for optimal activity [6,7]. Dz10-23 possess significant therapeutic potential due to their ability to cleave RNA involved in the pathogenesis of various diseases such as viral RNA [8], the mRNA of disease-related proteins [9,10] or regulatory non-coding RNA [11]. However, the effectiveness of Dz10-23 in vivo is limited due to the insufficient concentration of divalent cations, particularly Mg^2+^. Therefore, modifications within the Dz10-23 catalytic loop (i.e., within bases, deoxyribose or internucleotide bond) that enhance activity in low concentrations of divalent cations are being sought.

Cyclodeoxypurines (cdPus), i.e., 8,5′-cyclopurine 2′-deoxynucleosides, are products of oxidative DNA damage generated by hydroxyl radicals. In the initial step, the hydroxyl radical abstracts the hydrogen atom from the C5’ of deoxyribose and generates the carbon-centred radical. Next, the C5’ radical attacks the double bond between N7 and C8 of the purine base (either adenine or guanine) with the formation of a C5’-C8 bond. Depending on the spatial configuration of the substituents (i.e., -H and –OH) around the chiral C5’, two stereoisomers of cdPu exist with the 5′*R* (*R*cdPu) or 5′*S* (*S*cdPu) absolute configuration [12,13]. The C5’-C8 bond in cdPus induces significant structural changes compared to canonical deoxynucleosides (-tides). This includes a change in the conformation of the sugar ring, deformation of the sugar–phosphate backbone and displacement of the nitrogen base. In addition, the presence of cdPus in the DNA double helix disturbs the stacking interactions of the neighbouring nitrogen bases [14,15]. cdPus are formed endogenously, and their number is positively correlated with the level of hydroxyl radicals. Therefore, environmental factors that enhance the formation of hydroxyl radicals (for example, ionising radiation used during radiotherapy) will also increase the level of cdPu lesions. It is estimated that the frequency of cdPus in genomic DNA is in the range of 0.1–1 in 10^6^ nucleotides [16]. The presence of cdPus in the genetic material inhibits DNA replication and gene transcription. This type of lesion is repaired exclusively by the nucleotide excision repair (NER) mechanism, and the transcription-coupled NER (TC-NER) subtype seems to play the dominant role. The repair of *R*cdPu lesions is more efficient than *S*cdPu, probably due to greater structural deformations in DNA caused by the *R* isomer [17,18]. 

**Figure 1:**
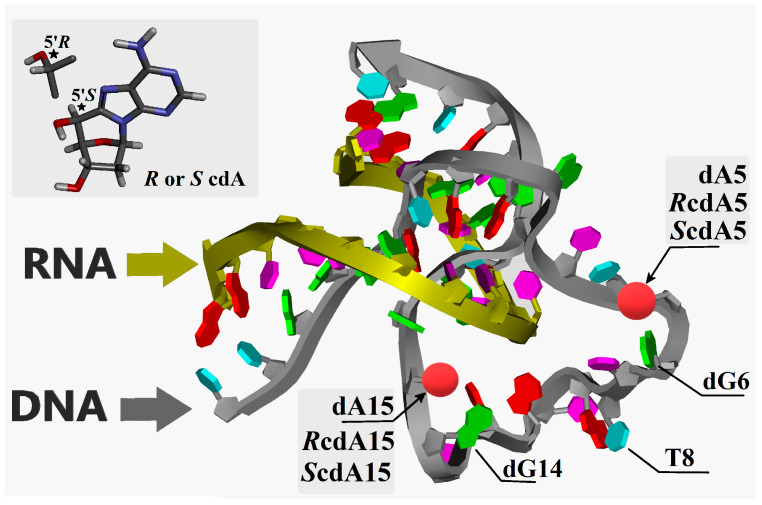
The spatial geometry of Dz10-23 with modifications indicated by red coloured balls, and the graphical representation of 5’S/R 5′8-cyclo-2’deoxyadenosine structures. Asterisk denotes a chiral carbon atom.

Since the development of Dz10-23 as a potential group of therapeutic nucleic acids three decades ago, many modifications have been introduced into their structure, such as inosine, 6-thioguanosine, deoxypurine, etc. [19]. In most cases, the flexibility of 2′-deoxyribose was not disturbed, except for in Lock Nucleic Acid derivatives, because of the presence of a methylene bridge. Moreover, the rotation of the nucleobase around the C1′-N9/N1 glycosidic bond was not perturbed in each of the discussed nucleoside analogues. In the present study, we investigated the effect of a DNA tandem lesion, i.e., stereo-defined 5′*S* or 5′*R* 5′,8-cyclo-2′deoxyadenosine (cdA), on the catalytic activity of Dz10-23 (Figure 1). cdA was introduced in position 5 or 15 of the catalytic loop, replacing the canonical dA. So far, the impact of cdA on the activity of Dz10-23 has not been investigated. Assuming the potential therapeutic application of DNAzymes, our studies may also reveal the possible limitations of the concurrent use of Dz10-23 and radiotherapy (i.e., ionising radiation), which may induce the conversion of dA(s) to cdA(s) and thus influence the activity of deoxyribozymes.

## 2. Results

### 2.1. 5′,8-Cyclo-2′deoxyadenosine (cdA) Doesn’t Alter the Stability of DNAzymes

**Figure 2:**
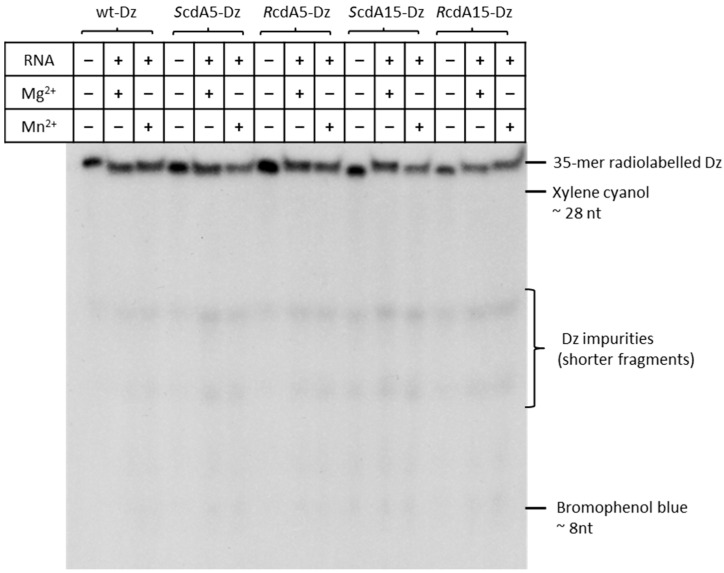
Polyacrylamide gel electrophoresis (PAGE) analysis of the effect of 5′-8-cyclo-2′deoxyadenosine (cdA) on the stability of Dz10-23. Positions of xylene cyanol (migrates as ~28 nt) and bromophenol blue (migrates as ~8 nt) are marked.

First, we checked whether the introduction of stereo-defined cdA modifications affects the stability of Dz10-23. P-32-labelled DNAzymes were incubated with substrate RNA and divalent cations (Mg^2+^ or Mn^2+^) for 1 h at 37 °C. The tests were carried out on unmodified Dz (wt-Dz) and its counterparts containing *R-* or *S-*cdA stereoisomers at positions 5 (i.e., *S*cdA5-Dz or *R*cdA5-Dz) or 15 (*S*cdA15-Dz or *R*cdA15-Dz) of the catalytic loop. As shown in Figure 2, the presence of cdA modifications had no effect on the stability of *S*cdA5-Dz, *R*cdA5-Dz, *S*cdA15-Dz and *R*cdA15-Dz.

### 2.2. Effect of cdA on the Catalytic Activity of Dz10-23 in the Absence of Divalent Cations

**Figure 3:**
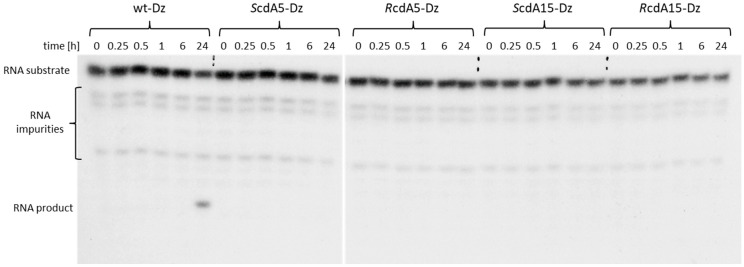
PAGE showing the effect of cycloadenosine in position 5 or 15 of the catalytic loop on the activity of Dz10-23 in the absence of divalent cations. RNA product corresponds to 11 nt fragment containing P-32.

We also examined whether cdA enhances the activity of Dz10-23 in the absence of divalent metal cations (Me^2+^). Canonical dA5 or dA15 in the catalytic loop were replaced by stereo-defined cdA (*R* or *S*). Catalytic activities of modified Dz10-23 were evaluated in the RNA cleavage assay and compared to the wt-Dz. The results presented in Figure 3 indicate that none of the cdA-modified Dz10-23 (i.e., *S*cdA5-Dz, *R*cdA5-Dz, *S*cdA15-Dz and *R*cdA15-Dz) showed any measurable catalytic activity within 24 h of incubation (no 11-mer RNA product detected). Only wt-Dz exhibited low residual catalytic activity detected after 24 h, probably due to the presence of trace amounts of Me^2+^ in the reaction buffer. 

### 2.3. Effect of cdA on Catalytic Activity of Dz10-23 in the Presence of Divalent Cations

**Figure 4:**
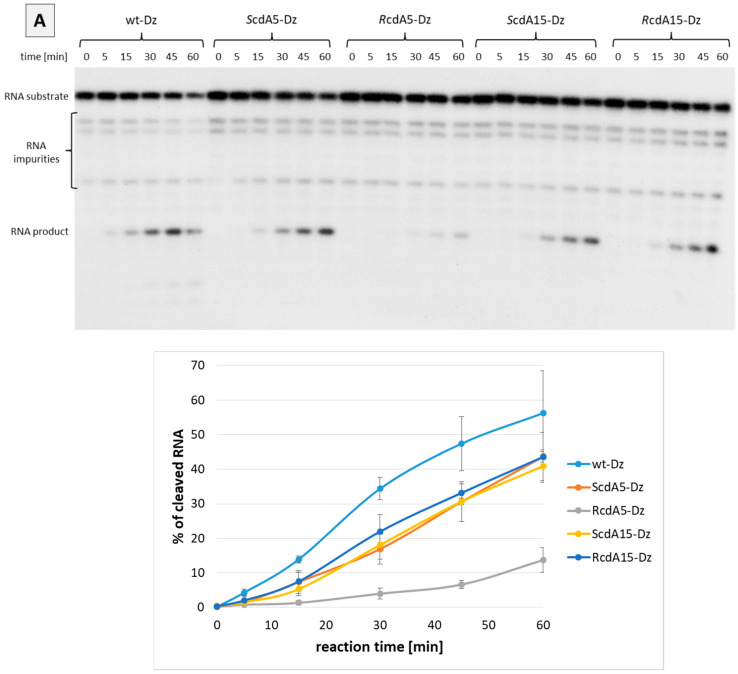

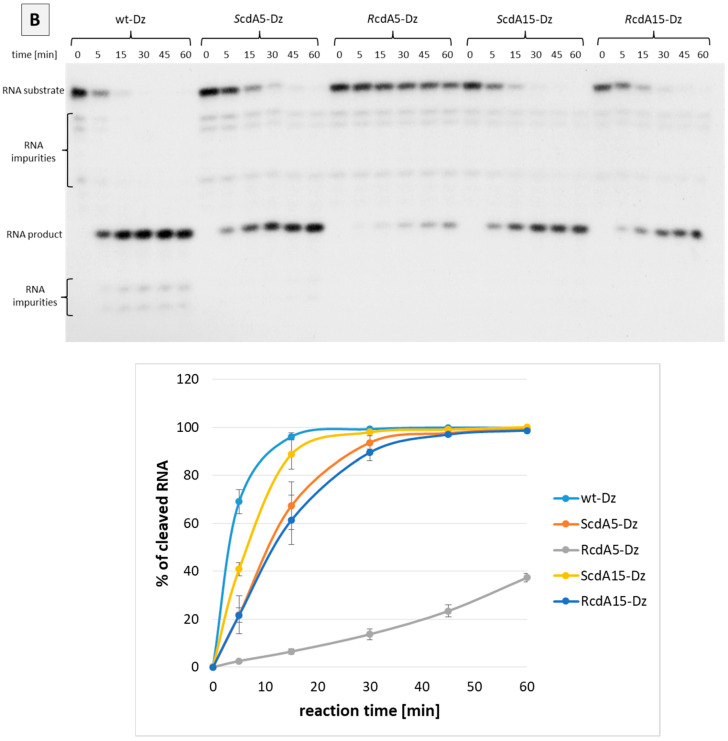
PAGE showing an effect of cdA on catalytic activity of Dz10-23 in the presence of divalent cations. (**A**) Mg^2+^, (**B**) Mn^2+^. The charts show relative amounts of RNA product (mean ± standard deviation (SD) from 3 independent experiments).

Next, we evaluated the effect of cdA modifications on the catalytic activity of Dz10-23 in the presence of Mg^2+^ or Mn^2+^. In general, the substitution of canonical dA with cdA in positions 5 or 15 of the catalytic loop decreased the activity of DNAzymes compared to the unmodified counterpart (wt-Dz). Interestingly, the level of inhibition was dependent on the position of cdA in the catalytic loop and its absolute configuration. For example, *S*cdA in positions 5 and 15 and *R*cdA in position 15 showed a modest inhibitory effect, while *R*cdA in position 5 strongly inhibited the activity of Dz10-23 (Figure 4A,B). This indicates that dA in position 5 of the catalytic loop is important for activity. It also suggests that *S*cdA and *R*cdA isomers in position 5 have different impacts on the structural organisation of the catalytic domain. The comparison of the activity of *S*cdA5-Dz and *R*cdA5-Dz clearly indicated that *R*-stereoisomer exerts a strong inhibitory effect, probably due to the conformational changes in the catalytic loop. A similar effect was observed irrespective of the divalent cations (Mg^2+^ or Mn^2+^) used as a cofactor of Dz10-23. It must be noted, however, that the catalytic activity of Dz10-23 was much higher in the presence of Mn^2+^ compared to Mg^2+^. This observation corroborates with previously published data, which suggest that Mn^2+^ has a higher binding affinity to Dz10-23. Another explanation points at the lower pKa of the manganese hydrate compared to magnesium [20].

### 2.4. Theoretical Studies

**Figure 5:**
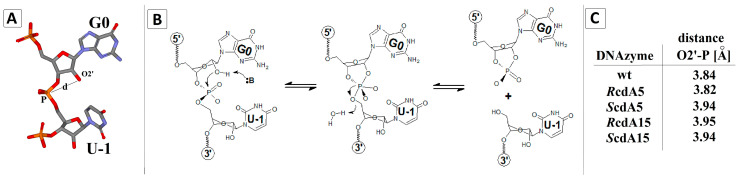
(**A**) The relative orientation of nucleotides G0 and U-1 of RNA in the catalytic site. (**B**) Graphical representation of the proposed RNA cleavage reaction by a Dz10-23. (**C**) The distances between 2’O of G0 and the phosphorus atom of the 3’ internucleotide bond in RNA, measured at ground state geometry, obtained on DFTB/3ob-3-1 level of theory in the aqueous phase.

The results of the biochemical experiments shown above revealed the following order of activity of DNAzymes: wt-Dz > ScdA5-Dz ≈ RcdA15-Dz ≈ ScdA15-Dz > *R*cdA5-Dz. Although the results were of interest, the reason for the observed effects could not be explained. With this in mind, we applied theoretical methods to shed light on the above cause. First, we investigated the geometry of the cleaved internucleotide bond in substrate RNA bound to wt-Dz and cdA-modified DNAzymes. Geometry optimisation with further molecular dynamics calculations in the aqueous phase was performed using Density Functional Tight Binding (DFTB) methodology with the self-consistent redistribution of Mulliken charge modification (SCC). Such a strategy is more accurate than a classical force field and less time-consuming than DFT. The cleavage of an RNA phosphodiester internucleotide bond requires a suitably positioned phosphorus atom, attached to the 5′OH group of U-1 and 2′O belonging to G0 (Figure 5A). A type 2 nucleophilic substitution is initiated by proton abstraction from the 2′ hydroxyl group by a base with a subsequent attack on the central phosphorus atom from the side of the highly negative substituent 5′ nucleoside, which, finally, after trigonal bipyramid reorganisation, leads to internucleotide bond cleavage (Figure 5B). Assuming that the starting geometry of the Dz10-23/RNA complex determines the cleavage reaction, we measured the distance between 2′O and P in the RNA substrate in the ground state (Figure 5C).

We also calculated and compared the overall spatial geometry of RNA/Dz10-23 complexes, using both wt-Dz and cdA-modified Dz. The structures were compared by the calculation of Root-Mean-Square Deviation (RMSD) values, as presented in Table 1.

Surprisingly, the obtained results did not indicate significant structural differences between *R*cdA5-Dz and wt-Dz. This is confirmed by the calculated distance between 2′O of G0 and 5′PO3 of U-1, i.e., 3.82 Å (*R*cdA5-Dz) versus 3.84 Å (wt-Dz), and the RMSD value of 0.238 Å^2^ for the DNA part and 0.261 Å^2^ for RNA.

Based on the above observations, it can be postulated that the introduction of 5′*R*cdA in position 5 scarcely changed the structure of DNAzyme, which is astounding given the obtained biochemical results. To explain this phenomenon, we analysed the distance between the 3′ and 5′ phosphate groups attached to the nucleoside at position 5 or 15 of Dz10-23. As shown in Table 2, at position 5, the greatest distance was calculated in the case of 5′*R*cdA (0.5Å greater than native dA and 0.3 Å greater than 5′*S*cdA). Similar results were obtained when 5′,8-cyclo-2′-deoxyadenosine was introduced at position 15 of Dz10-23. In this case, the differences between cdA and dA were 1.23 Å and 0.38 Å for 5′*R* and 5’*S* diastereomer, respectively. 

These differences in the distance between 3′ and 5′ phosphate groups can be directly accounted for by the structural rigidity of cdA, in which the rotation of the 5′ hydroxyl group around C4′-C5′ bonds and adenine around C1′-N9 bonds is prohibited. Furthermore, the pseudorotation cycle of the ribose five-membered ring was significantly reduced. All of the above led to the hindrance in the catalytic loop and a reduction in the activity of cdA-modified Dz10-23. 

**Figure 6:**
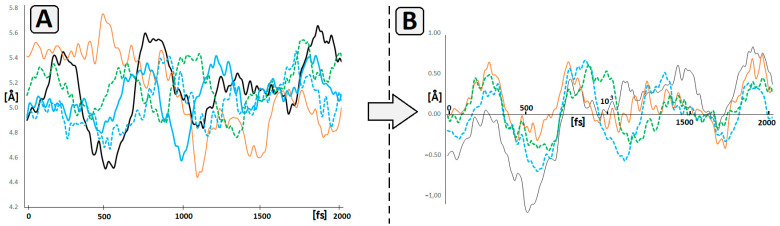
(**A**) Fluctuations of the distance (in Å) between phosphorus atoms of 5′PO_3_ and 3′PO_3_ groups attached to the nucleoside at position 5 of the catalytic loop of Dz10-23 (**^___^**wt, **^___^***R*cdA5, **^___^***S*cdA5, **---***R*cdA15, **---***S*cdA15), (**B**) The differences in P-P distance (in Å) between wt-Dz and **^___^***R*cdA5, **^___^***R*cdA15, **---***S*cdA5, **---***S*cdA15 as a function of time (raw MD data are presented in Supplementary Table S1).

A subsequent MD simulation revealed significant differences in 5’P-dN5-3’P distance as a function of time, between 5′*R*cdA5 and dA5, as shown in Figure 6A. The profiles of distance changes calculated for other DNAzymes were similar to wt-Dz. Based on these profiles, we also calculated the number of coincident points between wt-Dz- and cdA-modified DNAzymes. The coincident points corresponded to the same geometry of the analysed structures and were calculated as a subtraction of 5′P-dN5-3′P distance measured in the wt-Dz from that in cdA-modified Dz10-23. The following numbers of coincident points were found: 9, 16, 30 and 34 for *R*cdA5-Dz, *S*cdA5-Dz, *S*cdA15-Dz and *R*cdA15-Dz, respectively (Figure 6B). This suggests that the presence of 5’*R*cdA at position 5 of the catalytic loop reduces its ability to adopt the spatial geometry preferred for catalysis as in the case of wt-Dz. This also corroborates with the obtained biochemical results. 

Additionally, the results of MD experiments support the observation that the presence of cdA in DNA forces strong structural distortion in its 5′-end direction (Figure S1), which is in good agreement with previous DFT studies [21]. 

**Figure 7:**
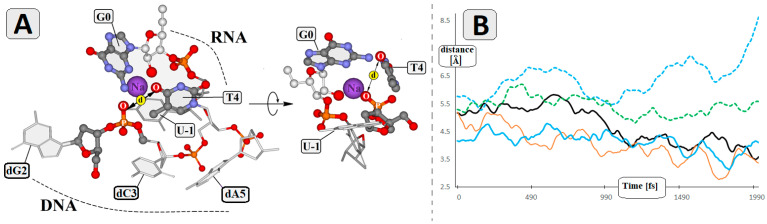
(**A**) Graphical representation of the Na ion (violet sphere) location at the catalytic site. (**B**) The distance fluctuations (in Å) between O4 of T4 and nonbonding oxygen of PO_2_ group attached to the 3′O of dG2 during 2000 fs period. Dz10-23: **^___^**wt, **^___^***R*cdA5, ^___^*S*cdA5, **---***R*cdA15, **---***S*cdA15 (raw MD data are presented in Supplementary Table S2).

Previous studies showed that the replacement of dG1, dG2, and dT4 by an AP site or C3 carbon linker did influence the RNA cleavage by Dz10-23, but only to a negligible degree. Conversely, the replacement of dT4 by another aromatic nucleobase ring forced a significant reduction in catalytic activity [22]. These results suggest that bivalent ions like Mg^2+^ are coordinated by phosphate groups present in this part of Dz10-23 [23]. Based on the above, we analysed the pre-catalytical spatial arrangement of G0, U-1, dG2, dC3 and dT4 in the context of sodium ion coordination using high-level SCC-DFTB methodology in the aqueous phase. The geometry analysis revealed that the Na^+^ is settled in the cage formed by G0, dT4 and the two phosphate groups between dG2-PO_2-_dC3 and G0-PO_2_-U-1. For the measurements, we arbitrarily chose the distance between O4 of dT4 and PO_2_ attached to 3′ hydroxyl of dG2 as an indicator of changes (Figure 7A).

In the case of RcdA5-Dz, the analysed distance adopted a value below that assigned for wt-Dz and as low as 2.81 Å. During the 2000 fs period, the investigated distance fluctuated between (in Å) 5.83 and 3.45, 5.14 and 2.81, 4.64 and 3.16, 6.21 and 4.88 and 8.63 and 5.28 for wt-Dz, RcdA5-Dz, ScdA5-Dz, ScdA15-Dz and RcdA15-Dz, respectively (Figure 7B). The above suggests that the 5′*R* diastereomer of cdA5 forces a structural effect (elongation) towards the 5′ end of the catalytic loop (dT4, dC3, dG2) and hinders Mg^2+^ penetration towards the site of SN_2_P reaction. This is confirmed in RcdA5-Dz, in which the distance between the two distal phosphate groups pertaining to the nucleoside at position 5 was 0.51 Å longer than that in wt-Dz (Table 2). Moreover, dT4 may act as a lid in the mentioned cage (Figure 7A), preventing Na^+^ exchange by Mg^2+^. 

## 3. Discussion

Our results support the notion that dA5 plays an important role in the activity of Dz10-23 [24,25]. Using the RNA cleavage assay, we demonstrated that the substitution of this canonical nucleoside with an *R*cdA stereoisomer resulted in the significant inhibition of RNA cleavage in the presence of Mg^2+^ or Mn^2+^ (i.e., 90% of inhibition compared to wt-Dz, reaction time 15 min). In contrast to *R*cdA5, substitution with an *S*cdA5 isomer resulted in the much weaker inhibition of activity compared to wt-Dz (i.e., 30–50% inhibition depending on the divalent cations, reaction time 15 min) (Figure 4A,B). This pronounced inhibitory effect of *R*cdA may be explained by structural changes within the catalytic loop, which may impair metal ion binding, or by conferring the rigidity of the catalytic loop. It has been reported that both diastereomers of 5′,8-cyclo-2′-deoxyadenosine force structural changes in DNA towards the 5’ end, depending on the 5′*R* or 5′*S* configuration [26]. Our theoretical calculations did not show significant structural changes evoked by RcdA5 with regard to the distance between 2′O of G0 and the phosphorus atom of the cleaved internucleotide bond in RNA (Figure 5C, Table 1). However, there are some differences, which may be considered responsible for the reduced catalytic activity. For example, the distance between phosphate groups at position 5 in RcdA5-Dz is significantly increased when compared to wt-Dz (Table 2). At the same time, the distance between O4 of dT4 and nonbonding oxygen of PO_2_ attached to 3′O of dG2 is much shorter (Figure 7). As a result, the flexibility of dT4 may be reduced, thus preventing the optimal positioning of Me^2+^ in the catalytic site. The above observations are in good agreement with Borggräfe et al., who mentioned in their NMR studies that *T4 undergoes an Mg^2+^-induced flip-out that may act as a molecular switch during activation* [7].

It has been postulated that the flexibility of the catalytic loop is crucial for catalysis [27]. However, cdA displays a very rigid structure, which was confirmed in NMR studies [28]. In cdA, the 2′-deoxyribose ring adopts an unusual West conformation _0_*E* [29] and the additional C5′-C8 covalent bond prevents the rotation of the 5′OH group around the C4′-C5′ bond as well as any rotation around the glycosidic bond C1’-N9 [30]. These features augment the inexorable structural changes induced by cdA towards the 5′ end of the DNA. Our results suggest that the introduction of RcdA5 into the catalytic loop may stiffen its structure and prevent from adopting the optimal catalytic conformation. This effect may be even more pronounced because the catalytic loop is a single-stranded DNA without complementary base pairs, which could reduce the distortion effect and stabilise the active structure of the DNAzyme. Altogether, the structural stiffness of the cdA can presumably be considered as a reason for the much lower activity of *R*cdA5-Dz than wt-Dz. Therefore, it is plausible that *R*cdA5 elicits structural disturbances that may hinder the stable coordination of Me^2+^ and lead to the inhibition of Dz activity.

We also demonstrated that dA15 is less relevant to Dz10-23 activity than dA5. The substitution of dA15 with *S*- or *R*cdA led to a moderate inhibition compared to wt-Dz. In contrast to cdA5, we did not observe significant differences in the activity of Dz containing *R*- or *S*cdA15. Our results are consistent with the previously published data, showing that dA15 together with dC13 and dG14 may be involved in the formation of a metal-ion-binding site located at the 3′ side of the catalytic loop. However, mutagenesis studies showed that while nucleobases dC13 and dG14 are essential for this activity, dA15 is less relevant [24]. The substitution of dA15 with dI (deoxyinosine), dG, dT and, to a lesser extent, with dC yielded active Dz, suggesting that dA15 is not directly involved in catalysis [25]. 

Interestingly, the degree of inhibition seems to depend on the type of divalent metal cations. In the presence of Mg^2+^, the activity of cdA-modified Dz10-23 was reduced by 50–65% compared to wt-Dz (reaction time of 15 min), while in the presence of Mn^2+^, the inhibition of activity was in the range of 10–40%.

Our studies also highlight the possible limitation of the concomitant use of therapeutic Dz10-23 and radiotherapy. In this case, ionising radiation may decrease the efficiency of Dz10-23 due to the induction of oxidative stress (i.e., hydroxyl radicals), which may subsequently induce the conversion of the catalytically relevant adenosine(s) to cycloadenosine(s).

## 4. Materials and Methods

### 4.1. Synthesis of Oligonucleotides

Oligonucleotides were synthesised and purified in the Dept. of Bioorganic Chemistry, Polish Academy of Sciences, Lodz, Poland. The synthesis was carried out on a Geneworld synthesiser (K&A Laborgeraete GbR, Schaafheim, Germany) using commercially available nucleosides phosphoramidites (ChemGenes Corporation, Wilmington, MA, USA). The phosphoramidite derivatives of cdA were synthesised as described by Romieu et al. [31]. The crude oligonucleotides were purified on HPLC (C-18 column, Synergi 4 μm Fusion-RP 80 Å, 250 × 4.6 mm, flow rate 1 mL/min) using Varian analytics with UV detection (λ = 260 nm). The following buffers were used: A) 0.1 M ammonium acetate (pH~7) and B) 40% CH_3_CN in buffer A. The HPLC purification gradient was as follows: from 0 to 35 min, the concentration of B increased from 0 to 100%, which remained on this level for 5 min with a subsequent decrease to 0% in 10 min; the equilibration time was 5 min. The concentration of oligonucleotides was determined by a Varian Cary 1.3E spectrophotometer (Varian, Brunn am Gebirge, Austria) from a measurement of maximum absorbance (λ = 260 nm).

Oligonucleotides were analysed on a Waters Synapt G2-Si HDMS quadrupole time of flight hybrid mass spectrometer (Waters, Manchester, UK) in the negative-ion mode. Samples were dissolved in 10 mM ammonium acetate with 50% acetonitrile to a concentration of 0.1 OD/mL. The analysis parameters were as follows: flow rate, 10 μL/min; capillary voltage, 2.6 kV; cone voltage, 40 V; the source temperature, 120 °C; the desolvation temperature, 400 °C; cone gas, 30 L/h; and desolvation gas, 600 L/h. The data were obtained in full-scan negative ion mode (mass range of 50–2000 *m*/*z*) and processed with Waters MassLynx 4.1 software (deconvolution with MaxEnt1 function, Waters Corporation, Milford, MA, USA). Mass data and mass spectra are shown in Supplementary Table S3 and Figure S3, respectively.

Sequences of Dz10-23 are shown below; the catalytic loop is underlined. cdA was introduced at positions 5 or 15 of the catalytic loop (bold, italic), and its absolute configuration (*R* or *S*) at C5′ of deoxyribose is also indicated.

5′ GAGTCCCATA GGCT***A***GCTACAACG***A*** AAGACTTGAG 3′; wt-Dz (unmodified).
5′ GAGTCCCATA GGCT ***R-cdA*** GCTACAACGA AAGACTTGAG 3′; *R*cdA5-Dz.
5′ GAGTCCCATA GGCT ***S-cdA*** GCTACAACGA AAGACTTGAG 3′; *S*cdA5-Dz.
5′ GAGTCCCATA GGCTAGCTACAACG ***R-cdA*** AAGACTTGAG 3′; *R*cdA15-Dz.
5′ GAGTCCCATA GGCTAGCTACAACG ***S-cdA*** AAGACTTGAG 3′; *S*cdA15-Dz.

Then, 21-mer synthetic RNA corresponding to the nucleotides 1245-1265 of β3 integrin mRNA (GenBank: J02703.1) was used as a substrate in the cleavage assay. The cleaved internucleotide bond is bolded. The 5′ end of substrate RNA was radiolabelled with ^32^P (asterisks) to enable autoradiographic detection and analysis of the 11-mer cleavage product.

5′ *CUC AAG UCU U**GU** AUG GGA CUC 3′; RNA substrate.

### 4.2. RNA Phosphorylation with γ(^32^P)-ATP

Phosphorylation of the 5′ end of RNA was performed as follows: samples (final volume 10 µL) containing 50 pmoles of RNA, 45 pmoles of γ(^32^P)-ATP (Hartmann Analytic, Germany, purity > 90%) and 5 units of T4 polynucleotide kinase (New England BioLabs, Ipswich, MA, USA; research grade) were incubated for 30 min at 37 °C in the T4-PNK phosphorylation buffer (New England BioLabs, Ipswich, MA, USA; research grade). Subsequently, radiolabelled RNA samples were denatured at 95 °C for 5 min and used for cleavage reactions catalysed by DNAzymes.

### 4.3. Phosphorylation of the 5′ End of Dz10-23

Samples (final volume 10 µL) containing 12.5 pmoles of Dz, 10 pmoles Y(^32^P)-ATP and 5 units of T4 polynucleotide kinase were incubated 1 h at 37 °C in the T4-PNK phosphorylation buffer. Next, samples were denatured at 95 °C for 5 min. 

### 4.4. Cleavage Assay

The reaction was carried out in the total volume of 15 µL at 37 °C for 0, 5, 15, 30, 45 and 60 min. The sample contained 25mM Tris pH 7.5 (GenoPlast Chemicals, Poland, purity > 99.9%), 25 mM MgCl_2_ (J.T. Baker, Philipsburg, NJ, USA, purity > 99.9%) or MnCl_2_ (Aldrich, Germany, purity > 98%), 0.01% of SDS (Fluka Biochemica, Buchs, Germany, purity > 98%), 12.5 nM of radiolabelled RNA and 12.5 nM of DNAzyme. The reaction was stopped by the addition of 3.5 µL of 0.5M EDTA (MP Biomedicals LLC, Illkirch, France, mol. biol. grade) and 9.5 µL of sample loading buffer (95% formamide (Sigma, Ronkonkoma, NY, USA, purity 99%), 0.025% bromophenol blue (MP Biomedicals LLC, research grade), 0.025% xylene cyanol (Sigma-Aldrich, Steinheim, Germany, research grade), 5 mM EDTA). Samples were immediately subjected to polyacrylamide gel electrophoresis or stored at −30 °C. The stability of the DNAzymes was evaluated in the same conditions except that radiolabelled DNA was used, and the reaction was carried out for 1 h.

### 4.5. Polyacrylamide Gel Electrophoresis

After 1 h of pre-electrophoresis, 8 µL of the samples was separated in 20% polyacrylamide gel containing 7M urea (Lach-Ner, Neratovice, Czech Republic, research grade) and 1× TBE (89 mM Tris, 89 mM boric acid (POCH S.A., Gliwice, Poland, purity not available), 2 mM EDTA, pH 8.2). Gels were exposed to X-ray films for 16 h at −30 °C, and the autoradiographs were visualised in a darkroom using standard developing and fixing chemicals (Foma Bohemia, Hradec Králové, Czech Republic).

### 4.6. Densitometry

X-ray films were analysed by densitometry using Quantity One 4.6.6 software (Bio-Rad, Hercules, CA, USA). The local background subtraction method was used for the calculation of band intensities.

### 4.7. Geometry Optimisation

In this study, the initial geometry of Dz10-23 was obtained by modifying the structure of 7PDU.pdb [7]; i.e., the cytidine at position 5 of the catalytic loop was replaced by 2′deoxyadenosine (denoted as wt-Dz), while the other oligonucleotides were left unmodified. Additionally, the negative charge of the phosphate group was quenched by sodium ions. The initial spatial geometry of dA5 was optimised using Density Functional Tight Binding (DFTB) methodology [32] with the self-consistent redistribution of Mulliken charge modification (SCC) [33]. The Third-Order Parametrization for Organic and Biological Systems with suspended Main Improvements Over parameter set was applied (noted as 3ob-3-1) [34]. Because of the complexity and size of the molecule structure, the Minnesota Solvation Model 12 (SM12) was used instead of a periodic box [35]. The generalised Born/solvent accessible surface area model (GBSA) of water with an angular surface grid of the solvent accessible 230 was applied. The convergence criterion for the SCC-DFTB interaction was set to ≤10^−5^ Hartree. Following the optimisation of the wt-Dz structure, the 2′-deoxyadenosine at position 5 or 15 of the catalytic loop was converted to 5′*R* or 5′*S* 5′,8-cyclo-2′-deoxyadenosine, and the modified Dz10-23 was noted as *R*cdA5, *S*cdA5, *R*cdA15 or *S*cdA15, as shown in Figure 1. The structures of the modified Dz10-23 were optimised at the same level of theory as have been described for the native wt-Dz. 

### 4.8. Molecular Dynamics

All the molecular dynamics calculations were performed at the SCC-DFTB/3ob-3-1 level of theory in the aqueous phase. The SM12 solvation model was used with 230 grids accessible. The SCC parameters were as follows: 500 interaction and 10^−8^ charge convergence. The previously optimised spatial geometries (ground state) of Dz10-23 dA5, *R*cdA5, *S*cdA5, *R*cdA15 and *S*cdA15 were utilised as the starting points of the MD calculations. All simulations were performed in the period of 2000 fs with a timestep of 0.25 fs, which gives 8000 points of trajectory with one of the sample frequencies. The isothermal-isobaric Nosé–Hoover chain (NHC) thermostat was used with the following parameters: 300 K as temperature, 1 fs of damping constant and an NHC chain length equal to 1 [36]. For all the theoretical geometry optimisations in this study, as well as molecular dynamics, the molecular ADF (Amsterdam Density Functional) program suit, version 2023.101 as a part of Software for Chemistry & Materials B.V. Amsterdam, Netherlands, was used [37].

## 5. Conclusions

The substitution of dA5 or dA15 by cycloadenosine decreased the catalytic activity of Dz10-23. The strongest inhibitory effect was observed for *R*cdA5, while *S*cdA5, *S*cdA15 and *R*cdA15 caused a moderate inhibition. Theoretical studies suggest that the incorporation of *R*cdA5 increases the rigidity of the DNAzyme, hampering the flexibility of catalytic loop, which may be required for the proper positioning of Mg^2+^ and optimal activity. Environmental factors (i.e., chemical agents, ionising radiation) that induce the formation of hydroxyl radicals may decrease the efficiency of Dz10-23 due to the conversion of the catalytically relevant adenosine(s) to cycloadenosine(s).

## Figures and Tables

**Table 1:** The structure overlapping expressed as RMSD of atomic positions in Å^2^, calculated for wt-Dz and its modified analogues, containing different diastereomeric forms of cdA. (The graphical representations have been given in the Supplementary Materials (SM), i.e., Figures S1 and S2).

Dz10-23	Spatial Geometry Comparison RMSD [Å^2^]	
	DNA Part	RNA Part
*R*cdA5 vs. wt	0.238	0.261
*S*cdA5 vs. wt	0.796	0.533
*R*cdA15 vs. wt	0.818	0.525
*S*cdA15 vs. wt	0.833	0.476

**Table 2:** The influence of cdA on the distance between phosphate groups of 5’P-dA5-3’P and 5’P-dA15-3’P. Measured distance (d) is illustrated in the graphical insert on the right.

**Dz10-23**	**Distance (d) [Å]**		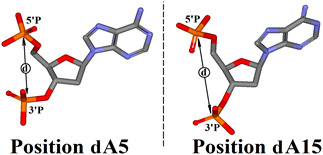
	**5′P-dA5-3′P**	**5′P-dA15-3′P**	
wt	4.90	5.91	
*R*cdA5	5.41	5.91	
*R*cdA15	4.94	7.14	
*S*cdA5	5.10	5.96	
*S*cdA15	4.92	6.29	

## Data Availability

Data contained within the article and Supplementary Materials.

## Supplementary Materials

The following supporting information can be downloaded at: https://www.mdpi.com/article/10.3390/ijms25052519/s1.
