# Supplementary material for: The Effect of 8,5′-Cyclo 2′-deoxyadenosine on the Activity of 10-23 DNAzyme: Experimental and Theoretical Study
Source: Int J Mol Sci. 2024 Feb 21;25(5):2519. doi: 10.3390/ijms25052519 (PMC10931185; doi:10.3390/ijms25052519)

Mass spectra of oligonucleotides.

RNA substrate

5' – CUC AAG UCU U**G**U AUG GGA CUC 3'; M(calc) 6650.018

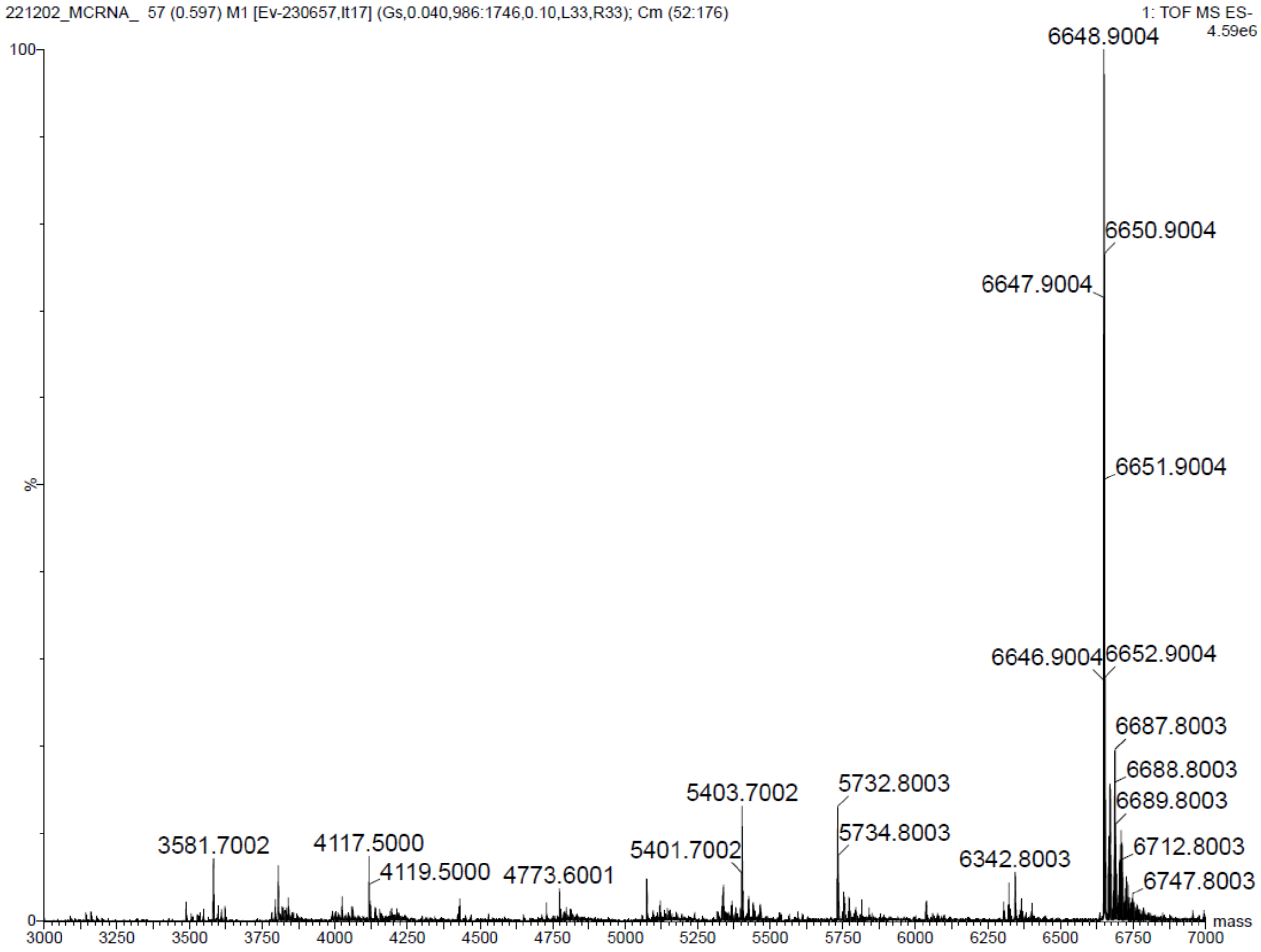

wt-Dz

5’ – GAG TCC CAT A <u>GG CTA GCT ACA ACG A</u> AA GAC TTG AG 3’; M(calc) 10798.107

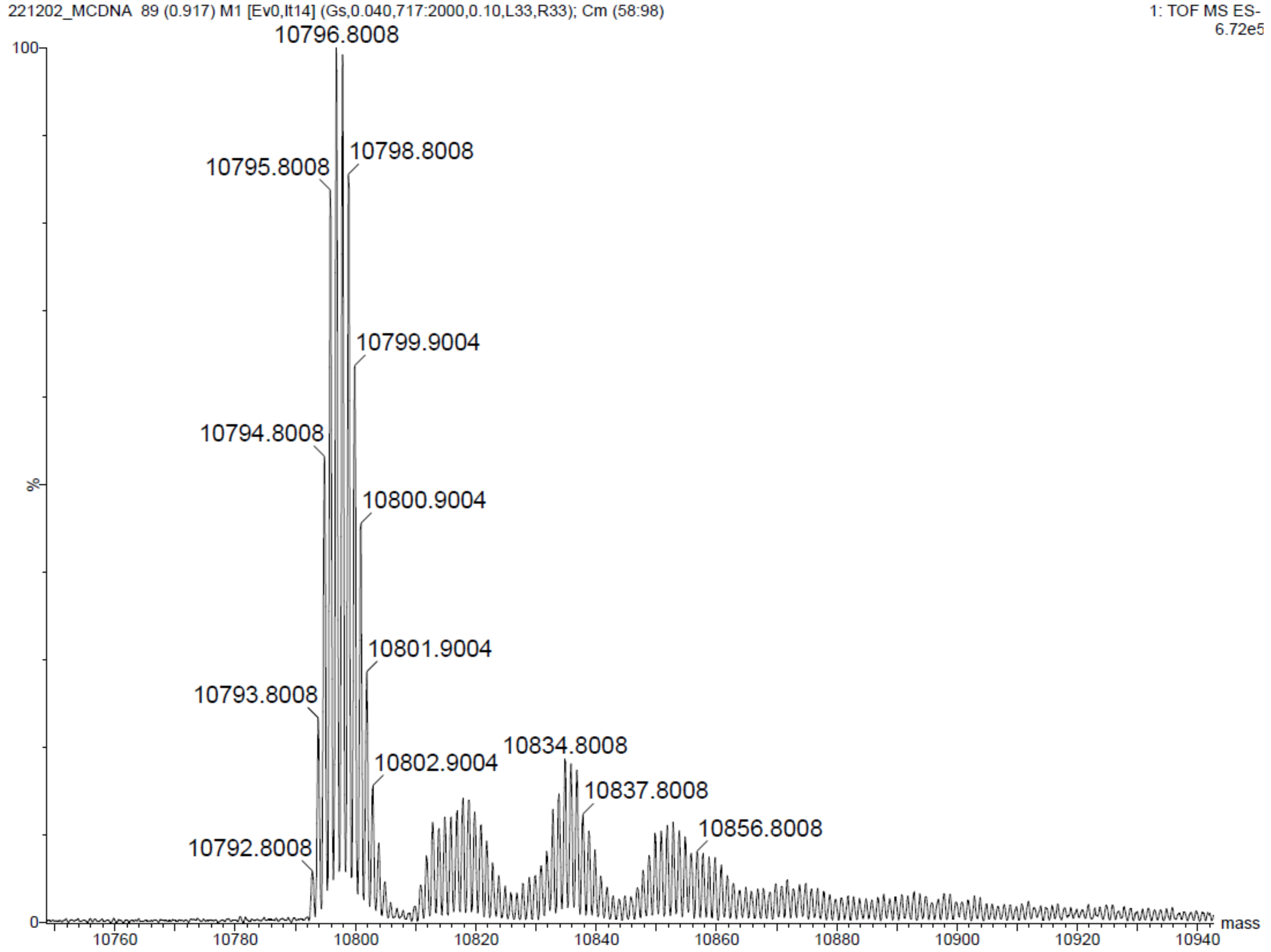

RcdA5-Dz

5’ – GAG TCC CAT A GGCT *R-cd*AGCT ACA ACGA AA GAC TTG AG 3’; M(calc) 10796.047

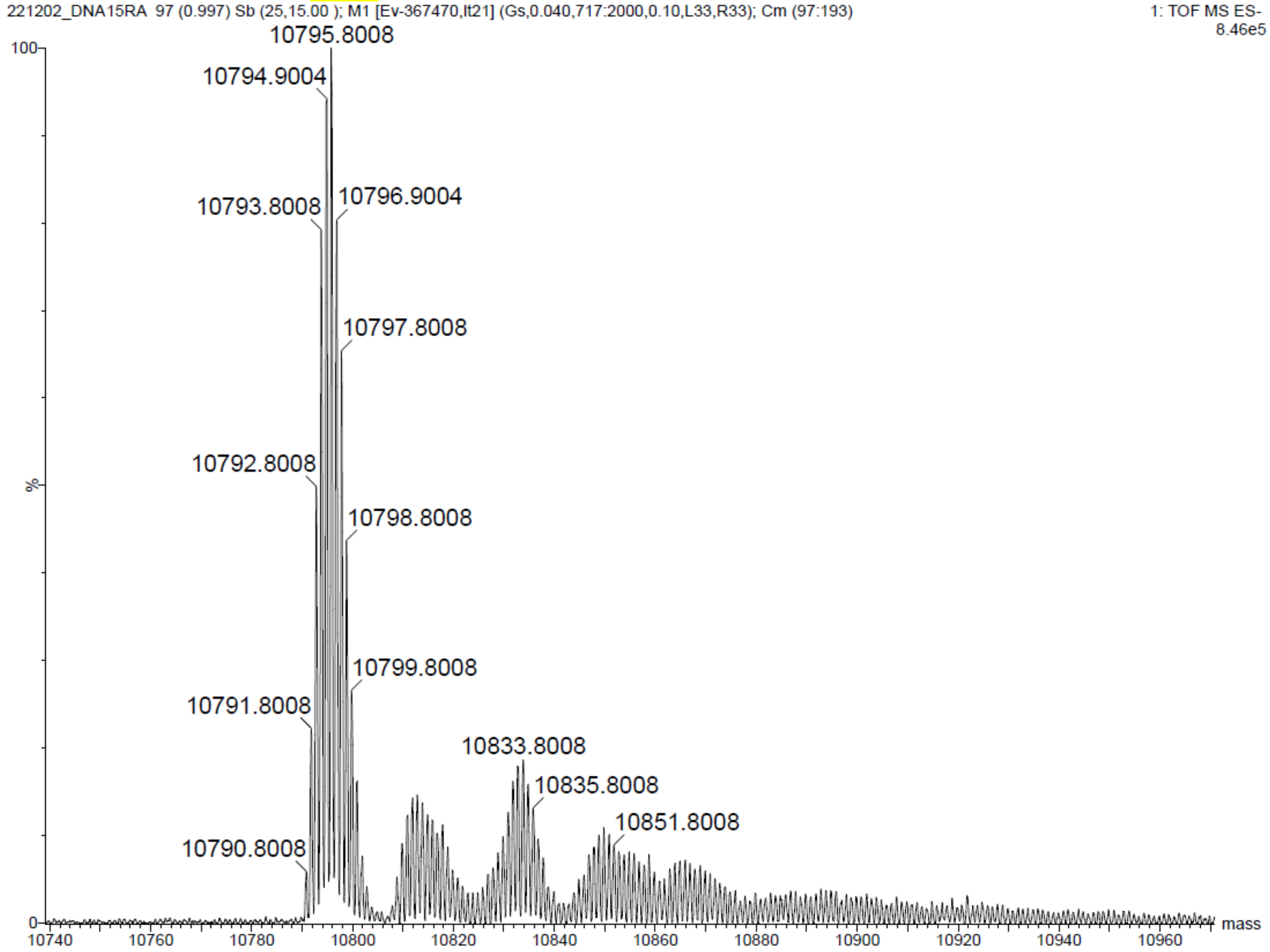

ScdA5-Dz

5' – GAG TCC CAT A GG CT *S-cd*AGCT ACA ACG A AAGAC TTG AG 3'; M(calc) 10796.047

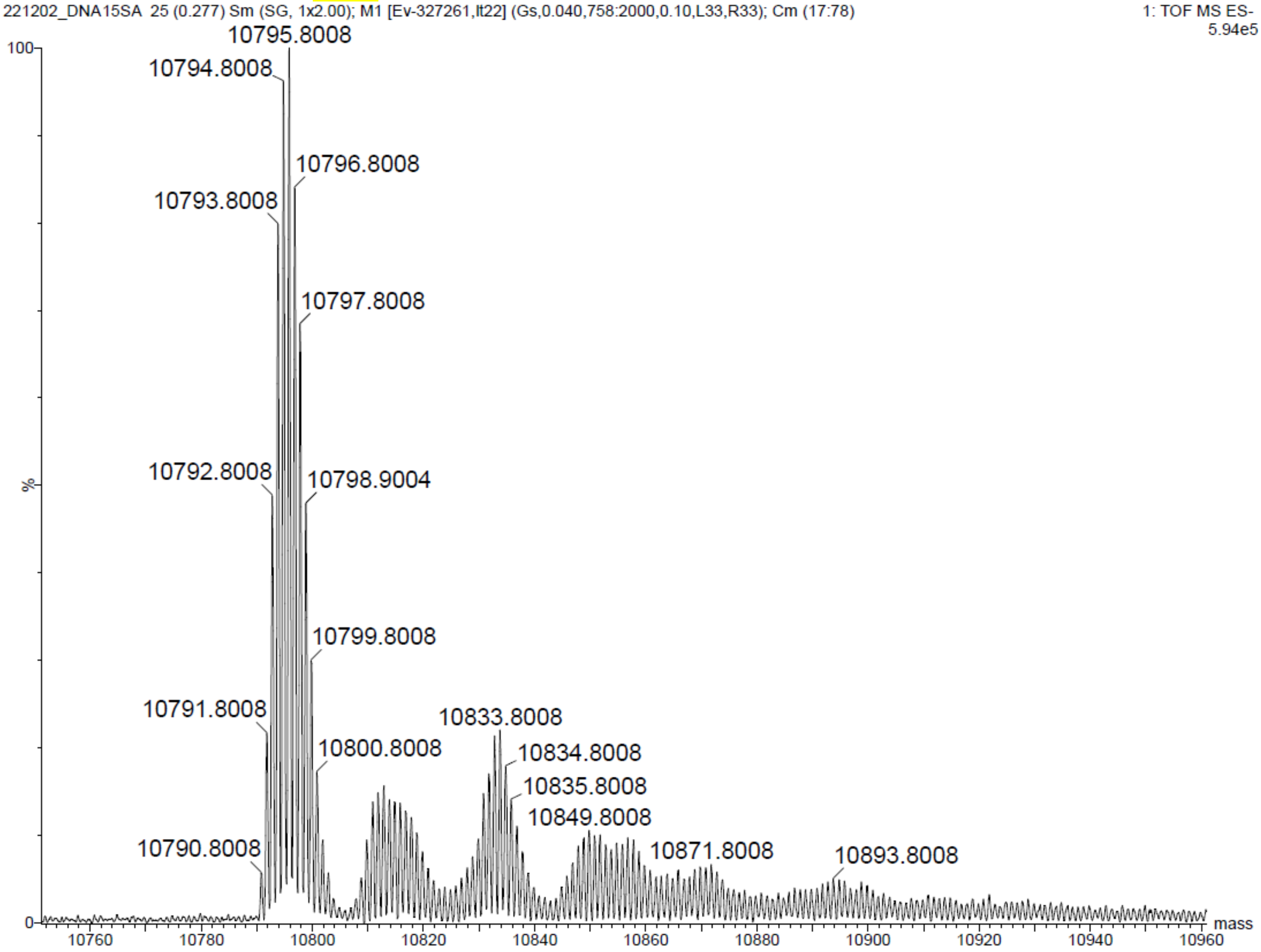

RcdA15-Dz

5’ – GAG TCC CAT A GG CTA GCTACA ACG *R-cdA* AA GAC TTG AG 3’; M(calc) 10796.047

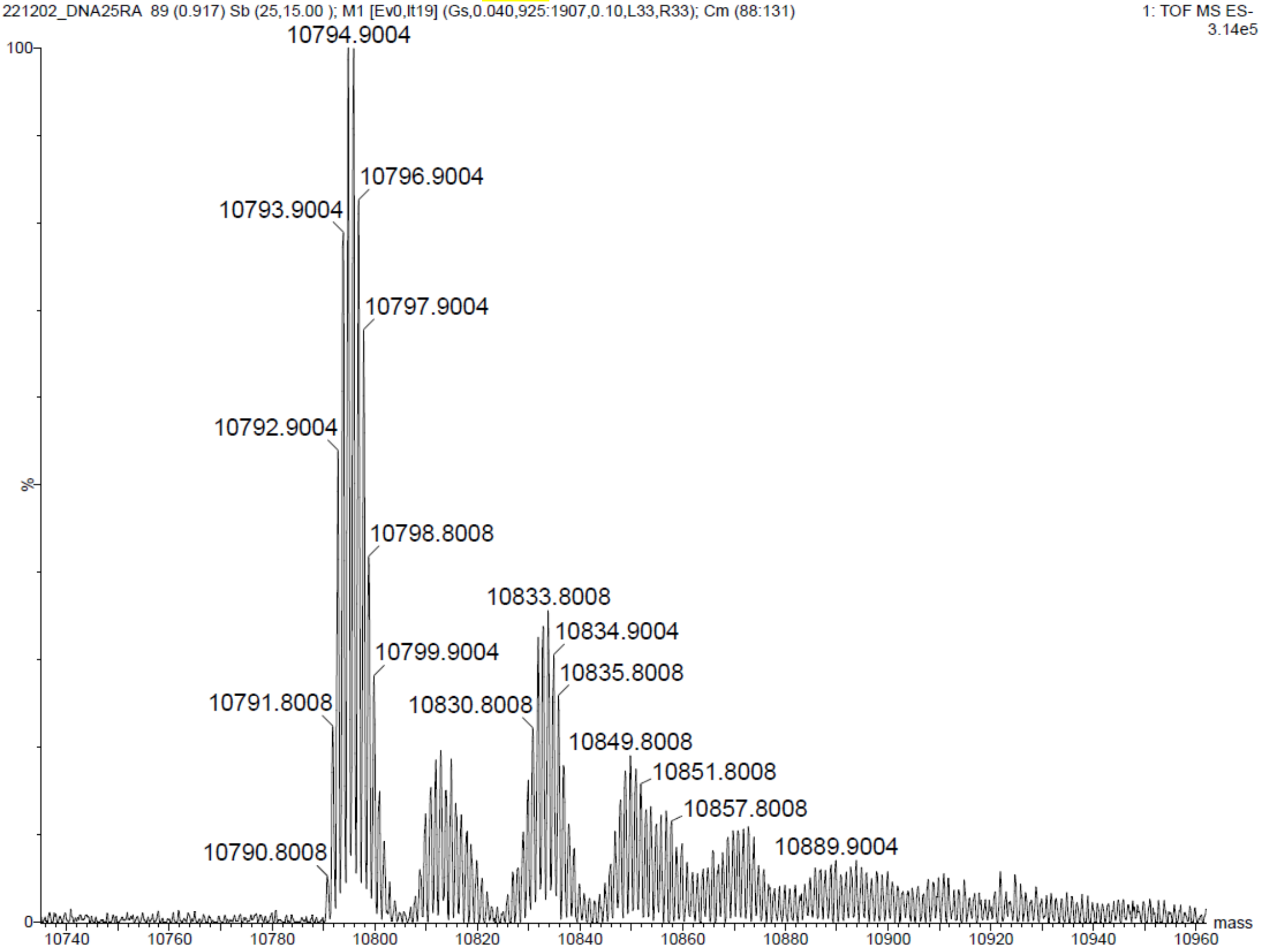

ScdA15-Dz

5’ – GAG TCC CAT A GG CTA GCTACA ACG *S-cdA* AA GAC TTG AG 3’; M(calc) 10796.047

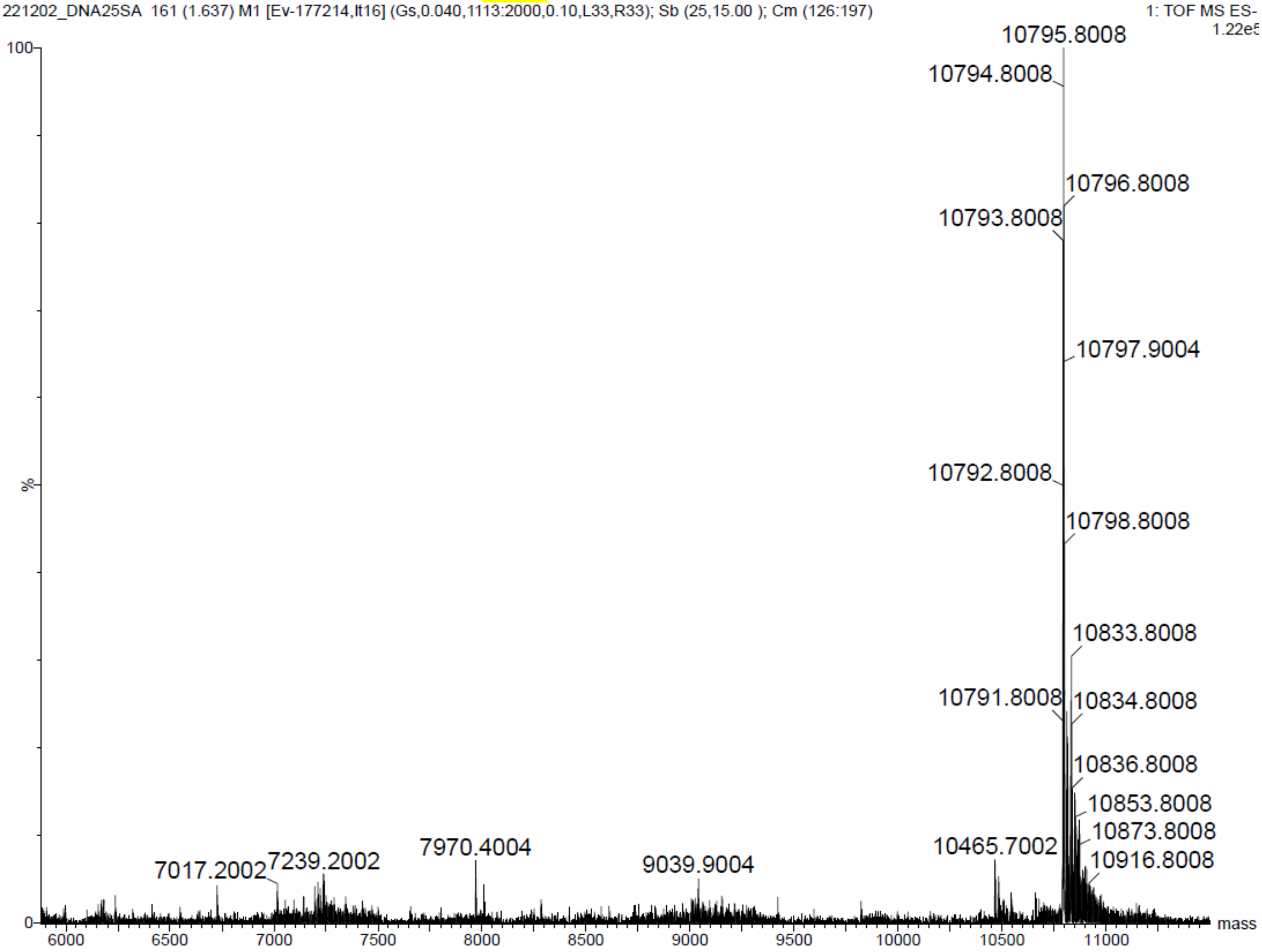

Supplement: Supplementary file 1 [file ijms-25-02519-s001.zip › Figure S3 Mass spectra.pdf]
