# Supplementary material for: The Effect of 8,5′-Cyclo 2′-deoxyadenosine on the Activity of 10-23 DNAzyme: Experimental and Theoretical Study
Source: Int J Mol Sci. 2024 Feb 21;25(5):2519. doi: 10.3390/ijms25052519 (PMC10931185; doi:10.3390/ijms25052519)
Supplement: Supplementary file 1 [file ijms-25-02519-s001.zip › Table S3 Mass data.pdf]

Oligonucleotide	Mass calc.	Mass found
wt-Dz	10798.107	10798.8008
5-R-Dz	10796.047	10795.8008
5-S-Dz	10796.047	10795.8008
15-R-Dz	10796.047	10794.9004
15-S-Dz	10796.047	10795.8008
RNA substrate	6650.018	6648.9004

RNA substrate
5’ – CUC AAG UCU U**G**U AUG GGA CUC 3’

wt-Dz
5’ – GAG TCC CAT A GG CTA GCT ACA ACG A AA GAC TTG AG 3’

5-R-Dz
5’ – GAG TCC CAT A GGCT *R-cd*AGCT ACA ACGA AA GAC TTG AG 3’

5-S-Dz
5’ – GAG TCC CAT A GG CT *S-cd*AGCT ACA ACG A AAGAC TTG AG 3’

15-R-Dz
5’ – GAG TCC CAT A GG CTA GCTACA ACG *R-cd*A AA GAC TTG AG 3’

15-S-Dz
5’ – GAG TCC CAT A GG CTA GCTACA ACG *S-cd*A AA GAC TTG AG 3’
