# Supplementary figures and images for: The Effect of 8,5′-Cyclo 2′-deoxyadenosine on the Activity of 10-23 DNAzyme: Experimental and Theoretical Study
Source: Int J Mol Sci. 2024 Feb 21;25(5):2519. doi: 10.3390/ijms25052519 (PMC10931185; doi:10.3390/ijms25052519)

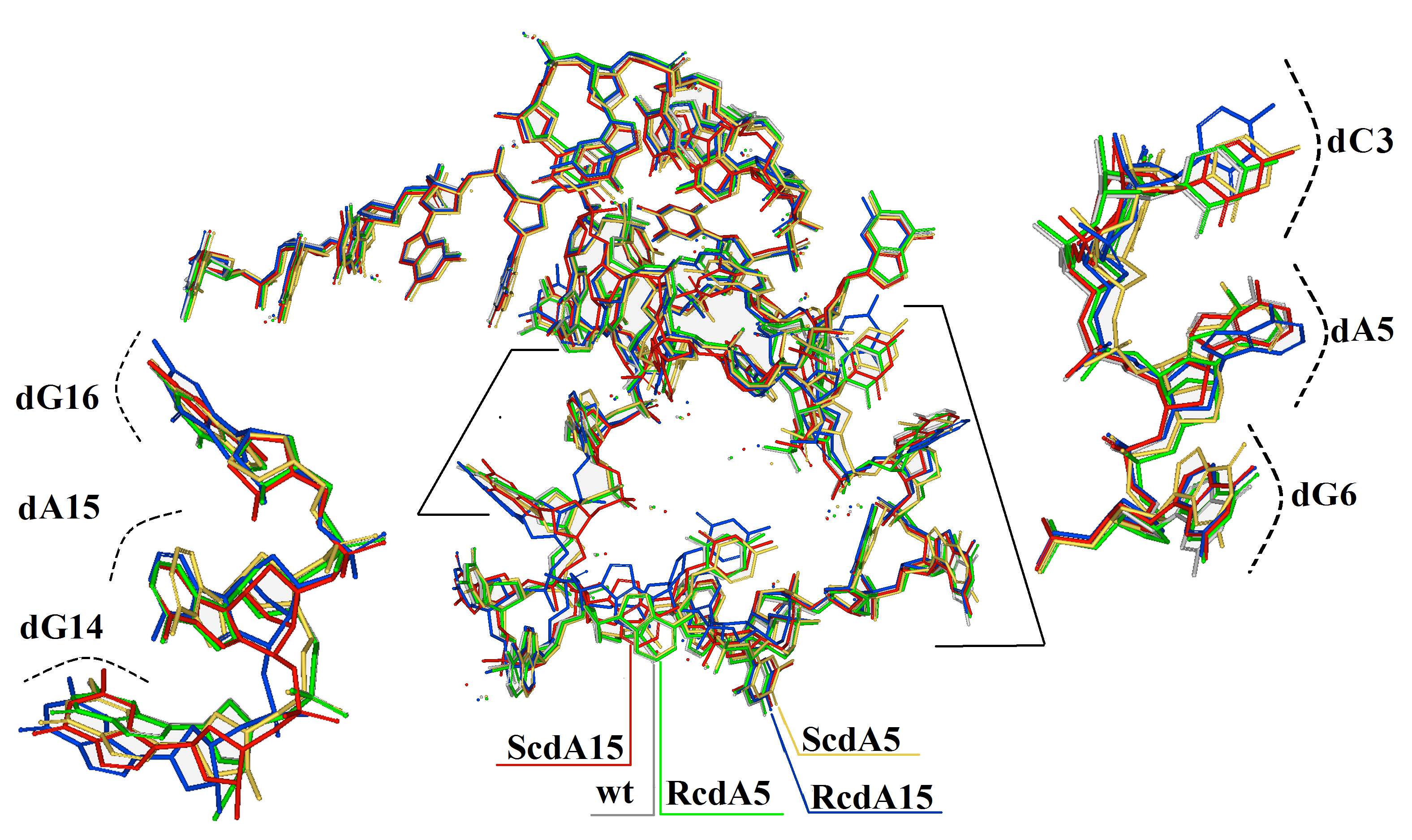

Supplement: Supplementary file 1 [file ijms-25-02519-s001.zip › Figure S1 (10_23DNAzyme DNA overalp).png]

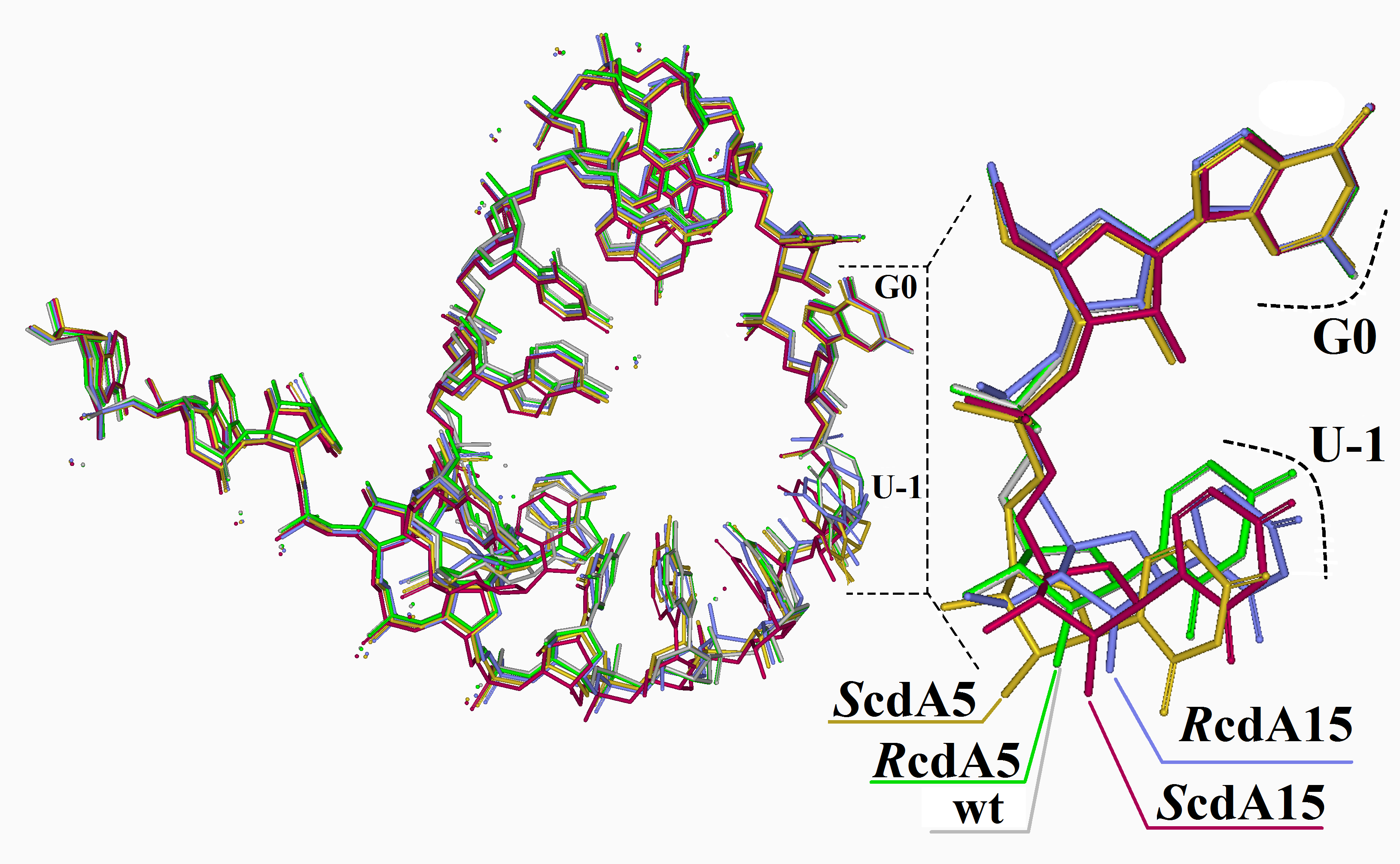

Supplement: Supplementary file 1 [file ijms-25-02519-s001.zip › Figure S2 (10_23DNAzyme RNA overalp).png]
